# High prevalence of baseline Non-R5 viral tropism in PLWH is associated with immune damage: a systematic review and meta-analysis

**DOI:** 10.3389/fimmu.2025.1701028

**Published:** 2025-12-02

**Authors:** Yangyang Liu, Defu Yuan, Yueqi Yin, Qian He, Meng Zhao, Hongfei Ma, Pingmin Wei, You Ge

**Affiliations:** 1 Department of Epidemiology and Health Statistics, School of Public Health, Southeast University, Nanjing, China; 2 Yinzhou District Center for Disease Control and Prevention, Ningbo, China; 3 School of Public Health, Xinjiang Medical University, Urumqi, China; 4 Department of Infectious Disease, the Second Hospital of Nanjing, Nanjing University of Chinese Medicine, Nanjing, China

**Keywords:** HIV-1, viral tropism, immune, CD4+T cell, epidemiology

## Abstract

**Objective:**

This systematic review and meta-analysis aims to characterize the distribution of HIV-1 viral tropism at diagnosis among people living with HIV (PLWH) and examine its association with baseline CD4^+^ T lymphocyte counts, thereby providing an evidence base for optimizing clinical interventions.

**Method:**

Observational studies reporting viral tropism prevalence and/or baseline CD4^+^ T cell counts stratified by tropism were retrieved from PubMed, Web of Science, Embase, and Cochrane Library. A random-effects model was employed for pooled prevalence estimation and mean difference calculation. Heterogeneity was quantified using I² statistics, with subgroup analyses and sensitivity tests to identify heterogeneity sources.

**Results:**

27 articles (N = 9372) were included in this study to analyze the distribution of viral tropism, and the prevalence of Non-R5 tropism was 15.68%. Subgroup analysis showed that the prevalence of Non-R5 IDU (27.86%) was significantly higher than that of sexual transmission (15.29%) and other routes (4.62%). The prevalence of Non-R5 tropism in the CRF01_AE subtype group (30.02%) was significantly higher than that of the B subtype (15.33%) and other subtypes (3.44%) (*P* ≤ 0.05). A comparison of CD4^+^ T cell counts (17 articles) showed a difference of −97.77 cells/μL for the Non-R5 tropic group relative to the R5 group.

**Conclusion:**

Our study find that PLWH with Non-R5 virus had more severe immune damage at diagnosis compared to PLWH with R5 virus. This can update the baseline status of patients in clinical practice. since this is a cross-sectional study, future cohort studies should be conducted to verify the relationship between tropism and changes in immunological indicators.

**Systematic Review Registration:**

https://www.crd.york.ac.uk/prospero/, identifier CRD420251088996.

## 1 Introduction

The human immunodeficiency virus (HIV) induces progressive immune destruction by infecting CD4^+^ T lymphocytes. HIV’s pathogenicity is closely associated with the interaction between viral envelope protein gp120 and host chemokine receptors (1). According to viral preference for chemokine receptor utilization, HIV-1 tropism is classified into three categories: 1) R5 tropic viruses that utilize C-C motif chemokine receptor type 5 (CCR5) for cellular entry (2), 2) X4 tropic viruses that utilize C-X-C motif chemokine receptor 4 (CXCR4) (3), and 3) R5/X4 tropic viruses capable of utilizing both CCR5 and CXCR4 receptors (4). HIV-1 transmitted/founder (T/F) viruses are almost exclusively R5 tropic and can efficiently infect CD4^+^ T cells with physiological CCR5 expression (5). In chronic progression, approximately 50~70% of people living with HIV (PLWH) experience viral tropism switching that results in X4 tropic viruses emergence. This process is typically associated with accelerated disease progression, CD4^+^ T cell depletion, and poorer clinical outcomes after antiretroviral treatment (ART) (6).

HIV tropism switching is recognized as a critical event in the natural history of infection, although the exact timing and driving mechanisms remain poorly characterized. Notably, some patients demonstrate completed tropism switching upon initial diagnosis. Current consensus indicates this phenotypic transition is associated with amino acid mutations in the V3 loop region (7). Viral chemokine receptor usage demonstrates subtype-dependent patterns: approximately 50% of subtype B infections develop X4 tropic variants (8), whereas CRF01_AE displays a higher prevalence of R5/X4 viruses (9). In contrast, Ndung’u et al. demonstrated that subtype C-infected PLWH rarely harbor R5/X4 tropic viruses at any disease stage (10). The population-level prevalence of R5/X4 tropic viruses has not been fully determined (11). Geographical variations in tropism conversion rates, which may reflect regional subtype distributions (e.g., subtype C predominance in Africa versus CRF01_AE prevalence in Southeast Asia), await systematic evaluation. Furthermore, epidemiological data concerning R5/X4 tropic virus prevalence at HIV diagnosis still require comprehensive synthesis.

Compared to R5 viruses, X4 tropic variants exhibit higher pathogenicity (12), and are associated with accelerated CD4^+^ T cell depletion in PLWH (11, 13). PLWH infected with X4 viruses show reduced sensitivity to ART (14). The impact of tropism on virological responses to ART remains controversial (15). CCR5 inhibitors serve as salvage therapy for R5 tropic infections (16), but become contraindicated upon emergence of R5/X4 tropic variants (11). Baseline CD4^+^ T cell count represents another critical determinant of ART outcomes, with higher baseline levels correlating with maximal potential for complete immune reconstitution (17). Elucidating both viral tropism and CD4^+^ T cell counts at diagnosis thus provides key evidence for formulating personalized antiretroviral therapy regimens and determining optimal timing for immune recovery interventions.

Given the lack of studies summarizing viral tropism characteristics at HIV diagnosis in PLWH, this study conducted a meta-analysis through systematic retrieval and integration of multicenter medical data. We updated the prevalence of R5 and Non-R5 tropism to 2025 and analyzed differences in baseline CD4^+^ T lymphocyte counts among PLWH infected with different tropism variants. By exploring the association between virological characteristics and immunological profiles at diagnosis, this work provides evidence-based guidance for optimizing the timing of ART and developing personalized immune reconstitution strategies.

Given the current lack of systematic studies on the prevalence of HIV viral tropism, this study aims to fill this gap. This study conducted a meta-analysis through systematic retrieval and integration of multicenter medical data. Our study extends the prevalence estimates of R5 and Non-R5 tropism through 2025, while examining baseline CD4^+^ T lymphocyte count disparities across tropism variants. Through elucidating virological-immunological correlations at diagnosis, this investigation offers evidence to refine ART initiation timelines.

## 2 Methods

### 2.1 Search strategy

The review protocol has been registered on PROSPERO (CRD420251088996). Data sources encompassed Web of Science, PubMed, Cochrane Library, Embase, and Scopus. Search terms included: HIV, human immunodeficiency virus, HIV-1, tropism, coreceptor usage, R5 tropic, X4 tropic, CCR5 receptor, CXCR4 receptor, CD4 Lymphocyte Count, CD4 count, CD4^+^ T cell, CD4 cell, and immune status. Prior to formal retrieval, all search terms were systematically searched within the MeSH thesaurus, supplemented with common expressions. The search timeframe spanned from the inception year of each database to November 4, 2025.

### 2.2 Inclusion/exclusion criteria

Inclusion criteria: 1) Cross-sectional studies published in English prior to November 4, 2025; 2) Participants were newly diagnosed, treatment-naïve HIV-1-infected individuals; 3) Studies reporting the number of participants stratified by viral tropism; 4) Sample size ≥ 20; 5) Additional criteria for CD4^+^ T cell analysis; 6) Reported CD4^+^ T cell count stratified according to viral tropism.

Exclusion criteria: 1) Non-original studies e.g., reviews, brief reports); 2) Participants aged < 15 years; 3) Pregnant or peripartum women; 4) Unclear methodology for viral tropism detection; 5) Viral tropism or CD4^+^ T cell count not assessed at diagnosis; 6) *In vitro*, animal, or mechanistic studies; 7) Insufficient data extraction or full-text inaccessibility. Only the earliest published studies were included for studies using the same cohort or data.

### 2.3 Data extraction and quality assessment

Literature management was conducted using Zotero 7 software. Data extraction and entry were independently conducted by two researchers using Excel software. Discrepancies were resolved through discussion until consensus was reached or by consulting a third author. The following information was extracted: first author, publication year, title, sample source, study period, sample collection, study population, number of R5 tropic and Non-R5 tropic infected individuals, and CD4^+^ T cell counts stratified by viral tropism. If studies included participants with distinct subtypes or genders and provided precise data, these subgroups were extracted as separate data points. Quality assessment criteria were adapted from the AHRQ guidelines for cross-sectional studies, incorporating the STROME-ID statement and study-specific characteristics, resulting in 11 assessment items for two predefined topics.

Quality Assessment Criteria (11 items): 1) Clearly defined study design (e.g., cohort study, cross-sectional study, or designs referenced to relevant protocols); 2) Specific inclusion and exclusion criteria; 3) Explicit statement of the study’s timeframe and sample sources; 4) Specification of sample types used in the study typically including plasma); 5) Clarification of the viral tropism detection methods employed; 6) Description of the threshold for genotypic prediction of tropism; 7) Documentation of the amplification success rate in the env region; 8) Description of any quality assurance tests performed; 9) Explanation of reasons for excluding study data or subjects; 10) Specification of measures to control potential laboratory contamination or confounding factors; 11) Explanation of strategies to address missing data in the analysis.

### 2.4 Subgroup settings

Gender: Male and Female; Area: China, European countries, and Other areas; Transmission route (Route 1): Sex, Inject Drugs Users (IDU), and Other. Sex transmission route (Route 2): MSM and Hetero; HIV-1 gene subtype: CRF01_AE; B Subtype and Non-CRF01_AE/Non-B’s other subtype; Recent infection: Unknown and Yes. Tropism detection: G2P and SVM; FPR threshold: 2% and 10%.

### 2.5 Meta-analysis and data statistics

Viral tropism was categorized into R5 tropic, X4 tropic, and R5/X4 tropic. Considering that in many studies, R5/X4 viruses exhibit characteristics of X4 viruses, the presence of R5/X4 viruses in an infected individual, like X4 viruses, may lead to the failure of CCR5 inhibitor therapy. X4 and R5/X4 viruses are combined as Non-R5 viruses. Accordingly, we also merged these two groups in this manner and established R5 virus and Non-R5 virus groups. In addition, only one included study reported R5/X4 virus data separately, and combining X4 and R5/X4 viruses also facilitated the analysis. Data management and cleaning were performed using Excel software, while statistical analyses were conducted with the meta package in R version 4.5.0. This study evaluated the pooled prevalence of Non-R5 viruses at HIV-1 diagnosis and the pooled effect size of CD4^+^ T cell counts between different tropism groups. If the distribution of rates does not follow a normal distribution, the arcsine square root transformation method is applied to make the data conform to a normal distribution. Heterogeneity was assessed using Cochran’s Q-test and I^2^ statistics. τ^2^ is more robust for small samples or extreme heterogeneity, avoiding negative variance estimates, and this statistic is used to jointly assess heterogeneity. Significant heterogeneity was defined as *P* ≤ 0.10 or I^2^ ≥ 25%. Random-effects models were employed when *P* ≤ 0.10 or I^2^ ≥ 50%; otherwise, fixed-effects models were applied. Publication bias was examined via Egger’s regression test (*P* > 0.05 indicating no substantial bias), with the trim-and-fill method used for adjustment if required. Sensitivity analysis was performed by sequentially excluding individual studies to assess result stability. Subgroup analyses were conducted to explore potential sources of heterogeneity.

## 3 Result

### 3.1 Study selection

A total of 3,079 references were retrieved from literature databases. After removing duplicates, 1,671 articles underwent title/abstract screening, with 1,137excluded (1,132 irrelevant to the topic; 5 non-English publications). Full-text review was conducted for 534 articles. A total of 507 articles were excluded based on predefined criteria: 116 irrelevant studies; 85 reviews/meta-analyses/guidelines/case reports; 7 involving pregnancy/perinatal women/vertically infected infants; 49 articles with participants aged <15 years; 8 case-control studies with uncalculable prevalence; 128 articles including antiretroviral therapy-experienced/undefined treatment status populations; 78 articles with non-newly diagnosed/undefined diagnosis status; 3 articles without baseline tropism testing; 25 articles with sample size <20; and 8 articles with unavailable data.

**Figure 1:**
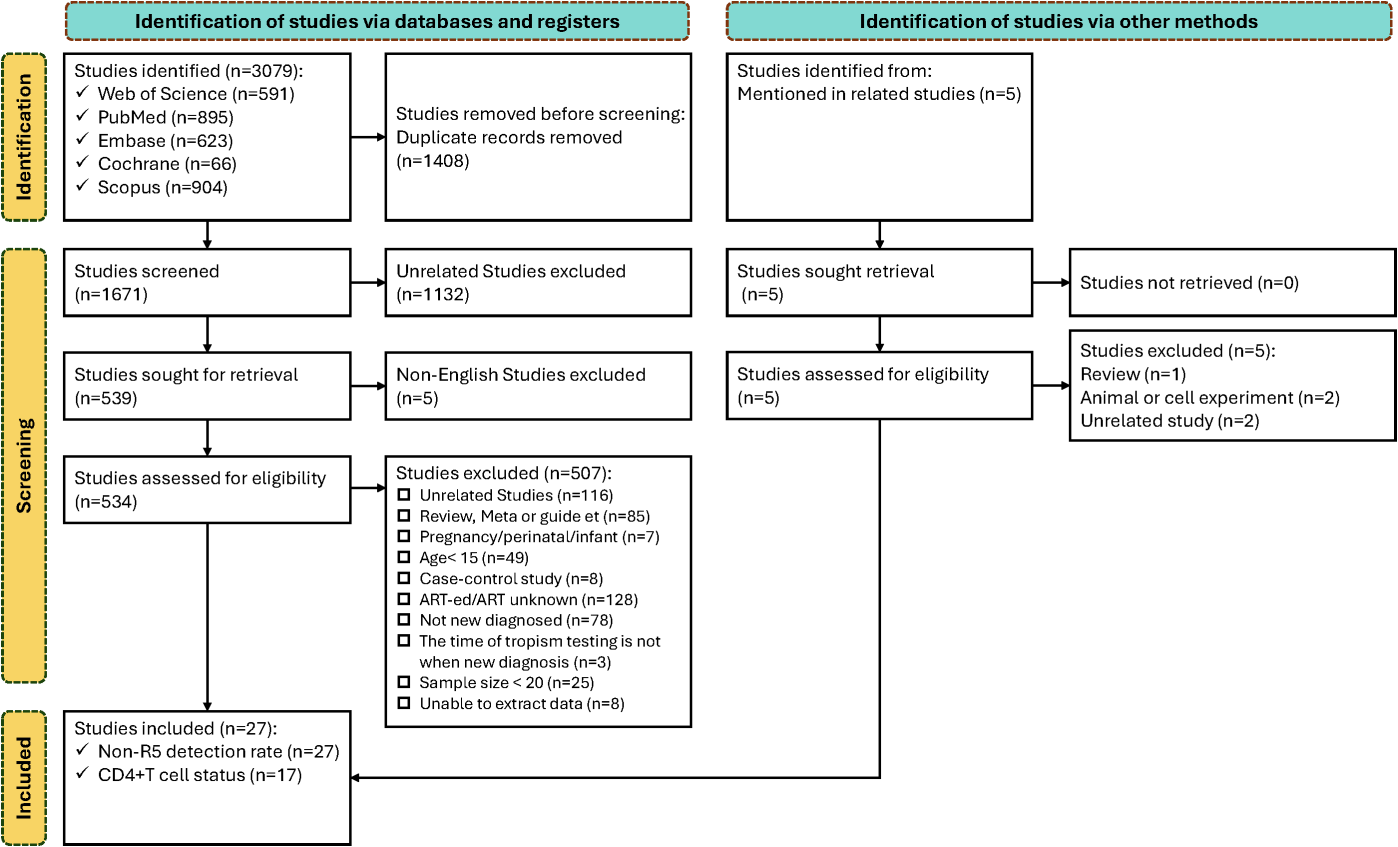
Flow chart of the study search and screening process.

27 articles were ultimately included in the analysis, among which 17 reported CD4^+^ T cell counts at diagnosis stratified by HIV tropism (R5 tropic vs. Non-R5 tropic). The study selection process is presented in **Figure 1**.

### 3.2 General characteristic

This study included 27 articles (27 data points) for meta-analysis of prevalence across different viral tropisms. The meta-analysis of CD4^+^ T cell count differences among individuals with distinct tropisms comprised 17 data points. **Table 1** presents characteristics of the 27 included studies, encompassing 9,372 HIV-1-infected individuals primarily distributed in China, France, Poland, and Spain. Three data points exclusively involved MSM populations, while the remaining data points were derived from general population studies. Quality assessment scores for each included study are provided in the **Supplementary Material**.

**Table 1:** Included study characteristics (N = 27).

ID	year	First Author	Sample Source	Subject	Number of		CD4^+^T Cell Count		Score
					Non-R5	R5	Non-R5	R5	
1	2024	Pang, X.	China	281	19	262	—	—	8
2	2024	Li, K.	China	48	16	32	123.94	407.44	8
3	2024	Dai, B.	China	152	18	134	—	—	9
4	2022	Hu, X.	China	2492	135	2357	292	380	7
5	2021	Lepore, L.	Italy	409	78	331	257	384.5	9
6	2020	Leda, A.R.	Brazil	24	5	19	168	327	9
7	2020	Connell, B.J.(a)	Netherlands	82	0	82	—	—	10
7	2020	Connell, B.J.(b)	South Africa	100	24	76	126	199	10
8	2019	Leone, A.	—	328	67	261	168	327	10
9	2019	Guo, J.L.	China	85	16	69	257	282	9
10	2015	Weichseldorfer, M.	China	108	28	80	316	348	9
11	2015	Parczewski, M.	Poland	194	54	140	—	—	8
12	2015	Dauwe, K.	Belgium	366	84	282	—	—	10
13	2015	Bon, I.	—	75	13	62	119	306	9
14	2014	Sierra Enguita, R.	Spain	737	145	592	574	602	10
15	2014	Li, Xiaoshan	China	276	72	204	—	—	8
16	2013	To, S.W. C.	China	191	74	117	—	—	10
17	2013	Mortier, V.	Belgium	225	36	189	—	—	10
18	2013	Frange, P.	France	555	27	528	422	525	10
19	2012	Soulié, C.	—	109	16	93	495	525	8
20	2011	Ghosn, J.	—	1387	202	1185	498	510	9
21	2010	Raymond, S.	France	125	8	117	482	481	8
22	2010	Demetriou, V.L.	Europe	152	5	147	—	—	9
23	2009	Huang, W.	United States	150	7	143	—	—	7
24	2009	Frange, P.	France	390	62	328	489	568	8
25	2008	de Mendoza, C.	Spain	61	10	51	450	629	9
26	2007	de Mendoza, C.	Spain	203	35	168	—	—	7
27	2007	Colome-Lluch, Marta	—	67	9	58	511	548	8

### 3.3 Tropism prevalence

**Figure 2:**
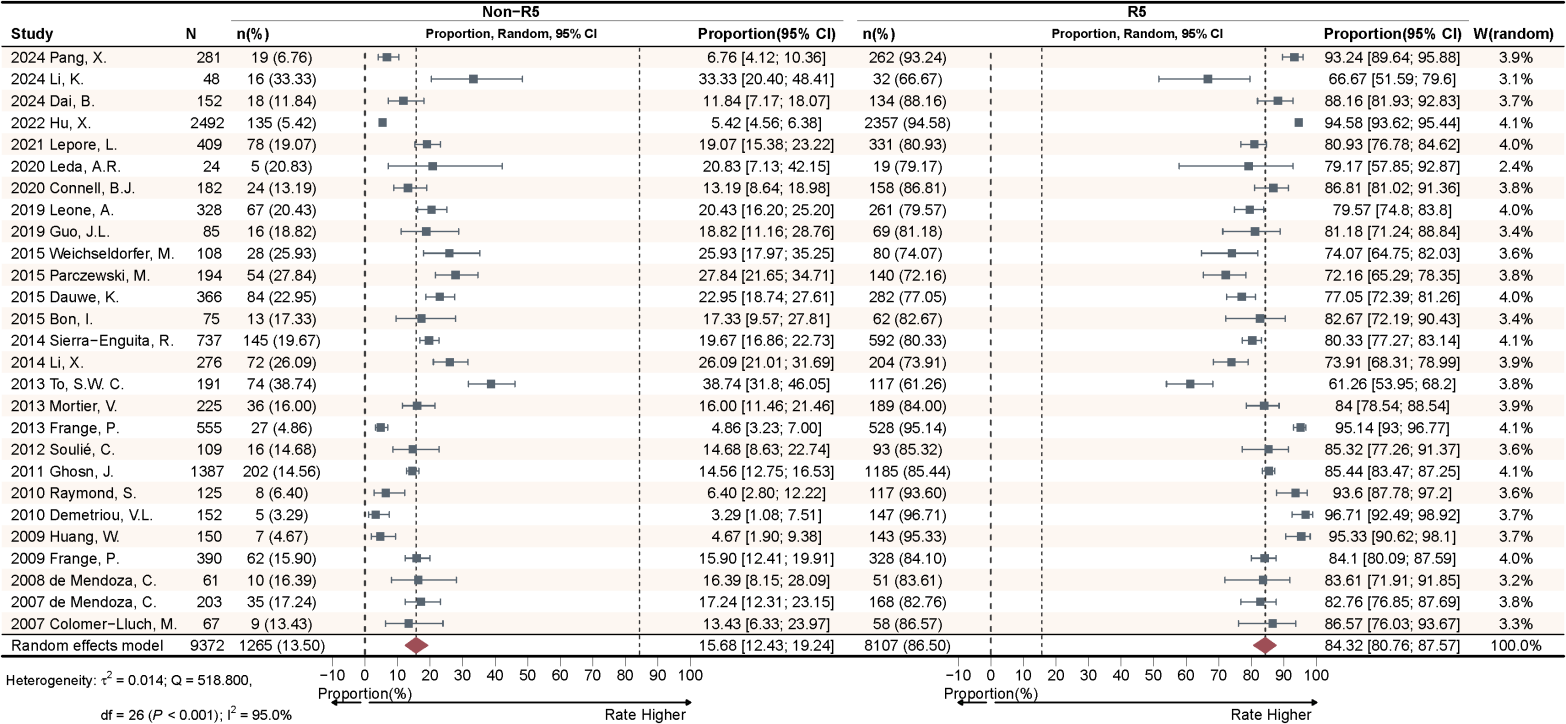
Meta-analyses on viral tropism prevalence among newly diagnosed PLWH.

This study included a total of 27 articles to calculate the pooled effect size of Non-R5 and R5 tropism virus prevalence among HIV-1 PLWH. The meta-analysis (**Figure 2**) demonstrated that the random-effects model yielded a pooled prevalence of 15.68% [95% confidence interval (CI): 12.43-19.24%] for Non-R5 tropism and 84.32% (95% CI: 80.76-87.57%) for R5 tropism. Substantial heterogeneity was observed with I²=95.0%, accompanied by significant between-study variation (Q_B_ = 518.800, *P* < 0.001).

### 3.4 Subgroup analysis

Area subgroup (23 data points): 1 article contributed 2 data points (Europe Country and Other), while 5 articles did not report sample sources. Transmission Route 1 subgroup: 6 articles included 2 data points (IDU and Sex), 2 articles reported 2 data points (Sex and Other), and 2 articles provided 3 data points (IDU, Sex, and Other). 12 articles lacked extractable subgroup information. Transmission Route2 subgroup (Sex transmission, 20 data points): 8 articles reported 2 data points (MSM and Hetero), and 15 articles lacked relevant data. HIV gene subtype subgroup (24 data points), 3 articles reported 3 data points (CRF01_AE, B, and other subtypes), 1 article provided 2 data points (CRF01_AE and B), 10 articles had no relevant data.

No statistically significant differences in Non-R5 tropism prevalence were observed among newly diagnosed PLWH across gender, geographic region, or Route2 subgroups (*P* > 0.05). However, significant differences were identified in Non-R5 tropism prevalence among PLWH stratified by Transmission Route1 and HIV gene subtypes (*P* ≤ 0.05) (**Table 2**).

**Table 2:** Subgroup analysis of the prevalence of PLWH virus tropism.

Subgroup	Study	Non-R5	R5	I^2^ (%)	Q_B_	*P*-value
Gender					0.16	0.688
Male	15	16.12 [12.11; 20.59]	83.88 [79.41; 87.89]	90.0		
Female	12	17.55 [12.34; 23.47]	82.45 [76.53; 87.66]	58.5		
Country/Area					1.58	0.454
China	8	19.12 [11.06; 28.78]	80.88 [71.22; 88.94]	97.3		
Europe Country	12	12.44 [7.23; 18.81]	87.56 [81.19; 92.77]	94.7		
Other	3	14.69 [3.94; 30.66]	85.31 [69.34; 96.06]	91.1		
Route1					11.30	0.004^*^
IDU	6	27.86 [19.55; 37.01]	72.14 [62.99; 80.45]	0.0		
Sex	15	15.29 [10.34; 21.01]	84.71 [78.99; 89.66]	91.4		
Other	5	4.62 [0.04; 16.20]	95.38 [83.80; 99.96]	83.4		
Route2					0.04	0.836
MSM	12	15.10 [8.68; 22.90]	84.90 [77.10; 91.32]	92.7		
Hetero	8	15.92 [13.19; 18.84]	84.08 [81.16; 86.81]	0.0		
Subtype					127.33	<0.001*
CRF01_AE	7	30.02 [15.53; 46.92]	69.98 [53.08; 84.47]	98.3		
B	14	15.33 [10.77; 20.54]	84.67 [79.46; 89.23]	28.1		
Non-AE/Non-B	3	0.11 [0.00; 0.36]	99.89 [99.64; 100.00]	87.3		
Tropism detection						
G2P	24	16.29 [12.76; 20.15]	83.71 [79.85; 87.24]	91.1	3.04	0.081
SVM	2	16.26 [12.11; 20.89]	83.74 [79.11; 87.89]	0.0		
FPR					1.63	0.202
2%	4	12.14 [3.82; 24.26]	87.86 [75.74; 96.18]	91.3		
10%	16	20.00 [16.50; 23.75]	80.00 [76.25; 83.50]	85.6		

*indicates statistically significant differences (*P* ≤ 0.05).

**Figure 3:**
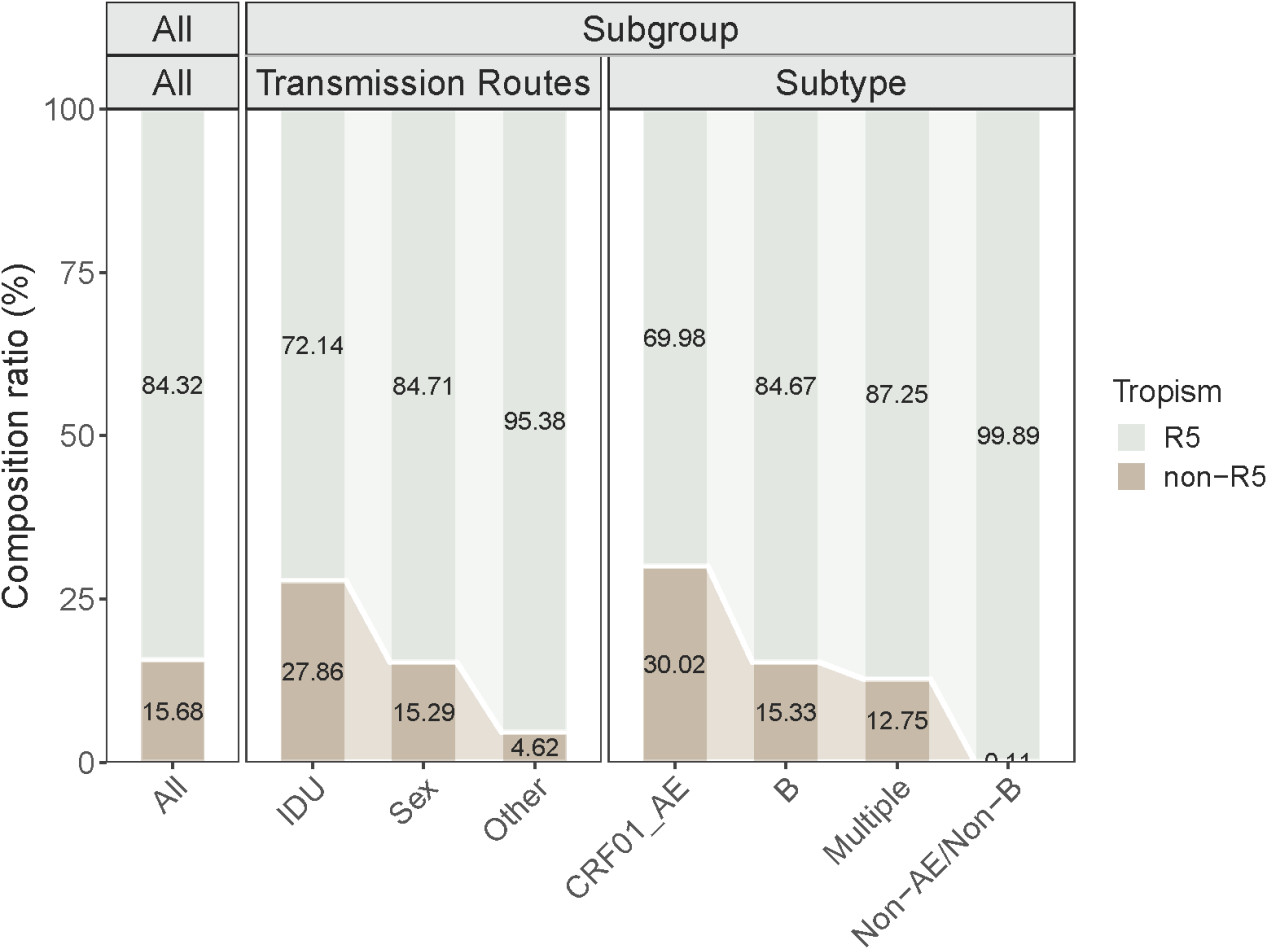
Characteristics of viral tropism in each subgroup.

**Figure 3** illustrates the prevalence of Non-R5 tropic and R5 tropic viruses across subgroups of PLWH at diagnosis. The Non-R5 tropic virus prevalence was significantly higher among IDU (27.86%) compared to those infected through sexual transmission (15.29%) and other routes (4.62%). In the CRF01_AE subtype group, the proportion of Non-R5 tropic viruses (30.02%) exceeded that observed in subtype B (15.33%) and other subtypes (3.44%). These inter-subgroup differences in tropism prevalence showed statistically significant variations (*P* ≤ 0.05).

### 3.5 Differences in immune characteristics

**Figure 4:**
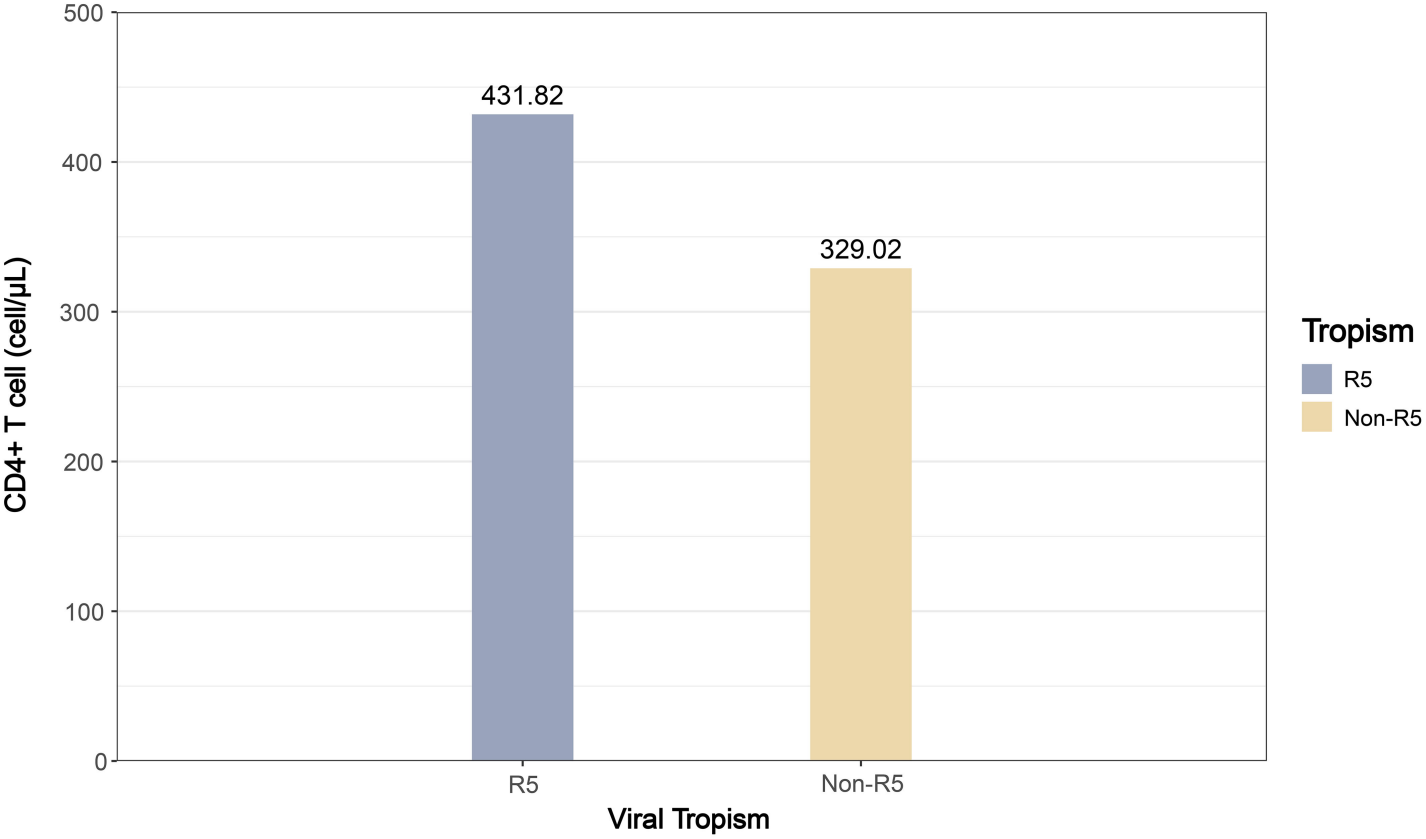
The bar plot of pooled effect sizes for CD4+ T cell counts by viral tropism (R5 vs. Non-R5) among PLWH.

**Figure 5:**
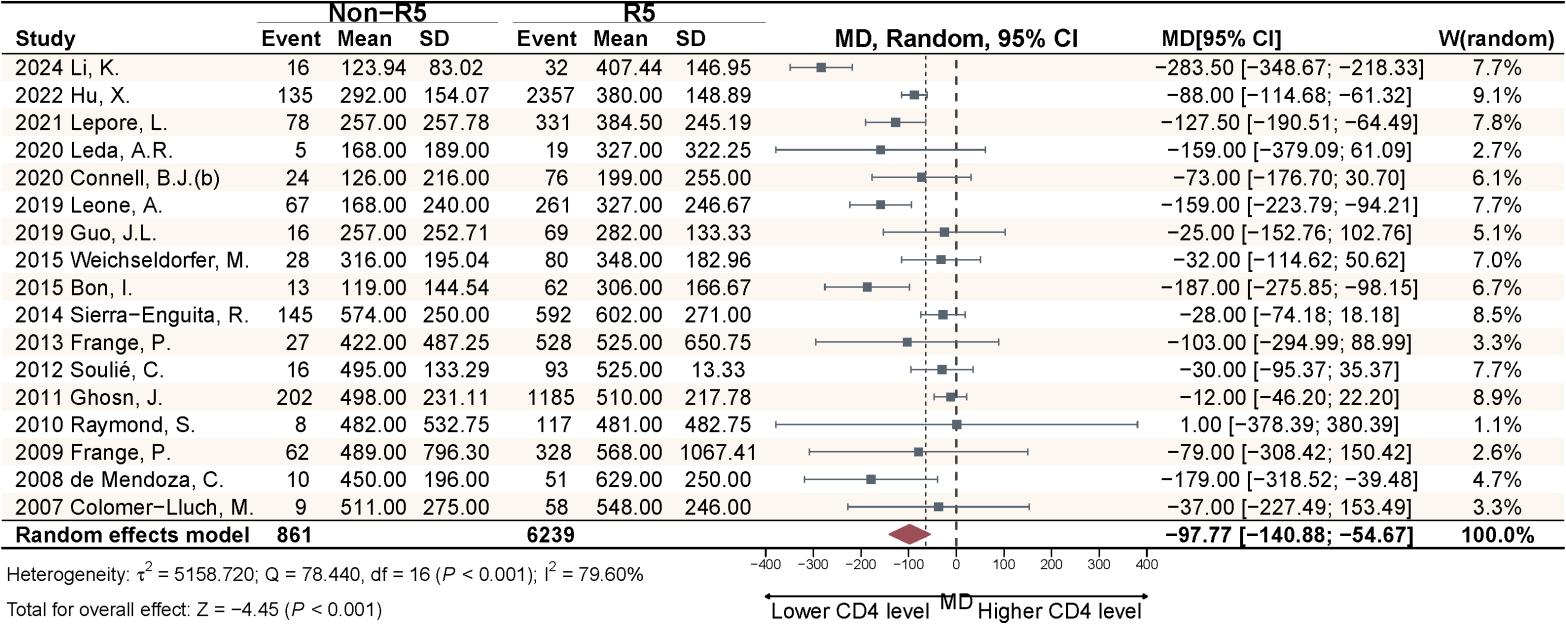
Meta-analysis of CD4^+^ cell count differences at diagnosis between viral tropism groups in PLWH.

This study included 17 articles comparing the pooled effect sizes of CD4^+^ T cell counts at diagnosis among PLWH with different viral tropisms. The pooled effect sizes of CD4^+^ T cells in PLWH with R5 tropic and Non-R5 tropic HIV were 431.82 and 329.02 cells/μL, respectively (**Figure 4**). The meta-analysis demonstrated that patients harboring Non-R5 tropic viruses had significantly lower CD4^+^ T cell counts at diagnosis compared to those with R5-tropic viruses, with a mean difference of −97.77 cells/μL (95% CI: -140.88 to -54.67) (**Figure 5**).

### 3.6 Publication bias and sensitivity analysis

This study constructed funnel plots and performed asymmetry tests to evaluate potential bias, with results presented in the **Supplementary Figures S1**, **S2**. Egger’s test indicated no significant publication bias for either component (*P*1 = 0.026; *P*2 = 0.431). To assess the robustness of our findings, we performed a leave-one-out sensitivity analysis. The results demonstrated that the pooled effect size remained stable without significant changes, indicating that our results are robust. For detailed results, please refer to **Supplementary Figure S3**.

## 4 Discussion

This meta-analysis and systematic review provides the most comprehensive contemporary evidence on HIV-1 tropism distribution among antiretroviral-naïve PLWH at diagnosis. Compared to previous comprehensive analyses, our study has updated through 2025 and incorporated published multicenter datasets with expanded geographic coverage. While focusing on inter-subtype differences in tropism prevalence, we have also conducted preliminary investigations into the association between viral tropism and pre-treatment immune status. The results demonstrate a Non-R5 viruses prevalence of 15.68% (95% CI: 12.43%-19.24%). Subgroup analysis revealed two distribution patterns: with a higher Non-R5 virus prevalence associated with IDU; marked inter-subtype variation in Non-R5 virus prevalence (CRF01_AE > B > Other subtypes). In addition, the significant difference in CD4^+^ T lymphocyte counts at diagnosis between Non-R5 virus carriers and R5 virus carriers (−97.77 cells/μL; 95% CI: −140.88 - −54.67) provides population-level validation.

This study revealed a 15.68% prevalence of Non-R5 viruses among newly diagnosed PLWH, suggesting these individuals may have passed the early/acute infection phase and represent advanced-stage diagnoses. Globally, late diagnosis rates among newly diagnosed patients ranges from 15.00% to 43.00% (18), with high-income countries reporting up to 50% late presentation (19). Specifically, European countries report rates of 22.20%~74.30%, Japan 71.12%, while recent data from Chinese indicate 57.60%~70.20% (20). These findings underscore the imperative to optimize diagnostic timelines, emphasizing enhanced screening coverage and innovative nucleic acid testing technologies for early detection. Timely diagnosis facilitates prompt ART initiation, a strategy endorsed by international consensus guidelines for its clinical benefits (19). Notably, expanded PrEP implementation has increased detection during seroconversion phase. Transient CD4^+^ T lymphocyte counts declines during seroconversion (21) may inflate late-presentation estimates. Comprehensive diagnostic evaluation integrating viral tropism assessment is therefore recommended to improve staging accuracy.

The subgroup analysis revealed a significant association between the IDU transmission route and higher prevalence of Non-R5 viruses (*P* = 0.004). This phenomenon may originate from the selective disadvantage of Non-R5 viruses in sexual transmission routes (22). Our study revealed that the prevalence of Non-R5 viral strains in CRF01_AE (30.02%) and subtype B (15.33%) was significantly higher than in other subtypes (3.44%). Li et al.’s (23) previously reported a 45.5% X4 viruses prevalence among treatment-naïve populations with CRF01_AE, while Zhang et al. (24) demonstrated an even higher X4 viruses prevalence of 61.30% in CRF01_AE. In contrast, X4 variants were virtually absent in CRF07_BC subtypes (0.00%). Notably, CRF01_AE has become the predominant subtype in Asia (84.00%) (25). Clinically, the 30.02% Non-R5 prevalence in CRF01_AE populations challenges CCR5 antagonists utility across East/Southeast Asia, while subtype B subtype’s 15.33% rate warrants cautious implementation in Western countries. prevalent regions. These geographically stratified findings underscore the necessity of tropism testing prior to coreceptor antagonist regimens, particularly in high-prevalence subtypes.

This study estimated the pooled effect size of baseline immune status across viral tropism groups. At diagnosis, PLWH with Non-R5 viral infections exhibited lower CD4^+^ T cell count (329.02 cells/μL) compared to those with R5 viruses (431.82 cells/μL), remained above 200 cells/μL. Conventional perspectives posit that Non-R5 tropic variants predominantly emerge during advanced disease stages (CD4<200 cells/μL) (26, 27). However, our findings revealed that tropism switching had already occurred when the disease had not yet progressed to the late stage. In conjunction with previous reports of higher Non-R5 prevalence in CRF01_AE and, these data suggest accelerated switching kinetics within this subtype, warranting further investigation into underlying drivers. The clinical implications of viral tropism characteristics are particularly relevant for treatment-experienced patients with potential multidrug resistance receiving CCR5 antagonists as salvage therapy, given their association with baseline immune profiles and therapeutic outcomes. Given that Non-R5 tropic variants predominantly emerge during advanced disease stages and considering the substantial proportion of late-presenters, this finding underscores the critical importance of early HIV detection for interrupting transmission chains and improving clinical outcomes. Based on these findings, it is recommended that clinicians implement more intensive immune monitoring for patients with Non-R5 tropic infections due to their accelerated progression to AIDS.

This study has several limitations. We were unable to incorporate data from ongoing investigations or unpublished sources, although publication bias assessment and sensitivity analyses were conducted. The insufficient data available to analyze tropism characteristics across additional subtypes and to establish further subgroup analyses examining the association between tropism and CD4^+^ T cell counts necessitate updates upon publication of future studies. Despite the non-significant findings in country/region subgroup analysis, the high representation of Chinese studies may introduce a regional selection bias, necessitating caution when generalizing conclusions to global context. Although subgroup analyses were conducted to attempt to address heterogeneity, there may remain unmeasured confounding variables or insufficiently reported data that precluded comprehensive analysis. The cross-sectional design of this study precludes establishing temporal associations between tropism and immunological parameters. Prospective longitudinal studies are warranted to elucidate these dynamics. These limitations underscore the necessity to update the analyses as more robust evidence emerges.

## 5 Conclusion

This study analyzed the distribution of viral tropism and its association with baseline immune status in newly diagnosed PLWH. providing critical evidence for optimizing clinical testing strategies. Non-R5 viruses were found to be prevalent among newly infected individuals, exhibiting subtype-specific distribution patterns. This study findings highlight the necessity of early detection of Non-R5 infections and provide critical evidence for guiding individualized clinical management. Future studies should validate these patterns across broader geographical regions and explore tropism evolution mechanisms associated with long-term immune reconstitution.

## Data Availability

The original contributions presented in the study are included in the article/**Supplementary Material**. Further inquiries can be directed to the corresponding authors.
