# Supplementary material for: High prevalence of baseline Non-R5 viral tropism in PLWH is associated with immune damage: a systematic review and meta-analysis
Source: Front Immunol. 2025 Dec 2;16:1701028. doi: 10.3389/fimmu.2025.1701028 (PMC12705401; doi:10.3389/fimmu.2025.1701028)
Supplement: Supplementary file 1 [file Table1.docx]

Supplementary Material

# This Word file includes:

Description of supplemental figures

Supplementary Figure S1 to S3

**Supplementary Figure S1:** Publication bias assessment for the meta-analysis of virus tropism prevalence at diagnosis among people living with HIV (PLWH).

**Supplementary Figure S2:** Publication bias assessment for the meta-analysis of immunological correlates of HIV tropism at diagnosis in people living with HIV (PLWH).

**Supplementary Figure S3:** Forest plots of the leave-one-out sensitivity analysis.

**
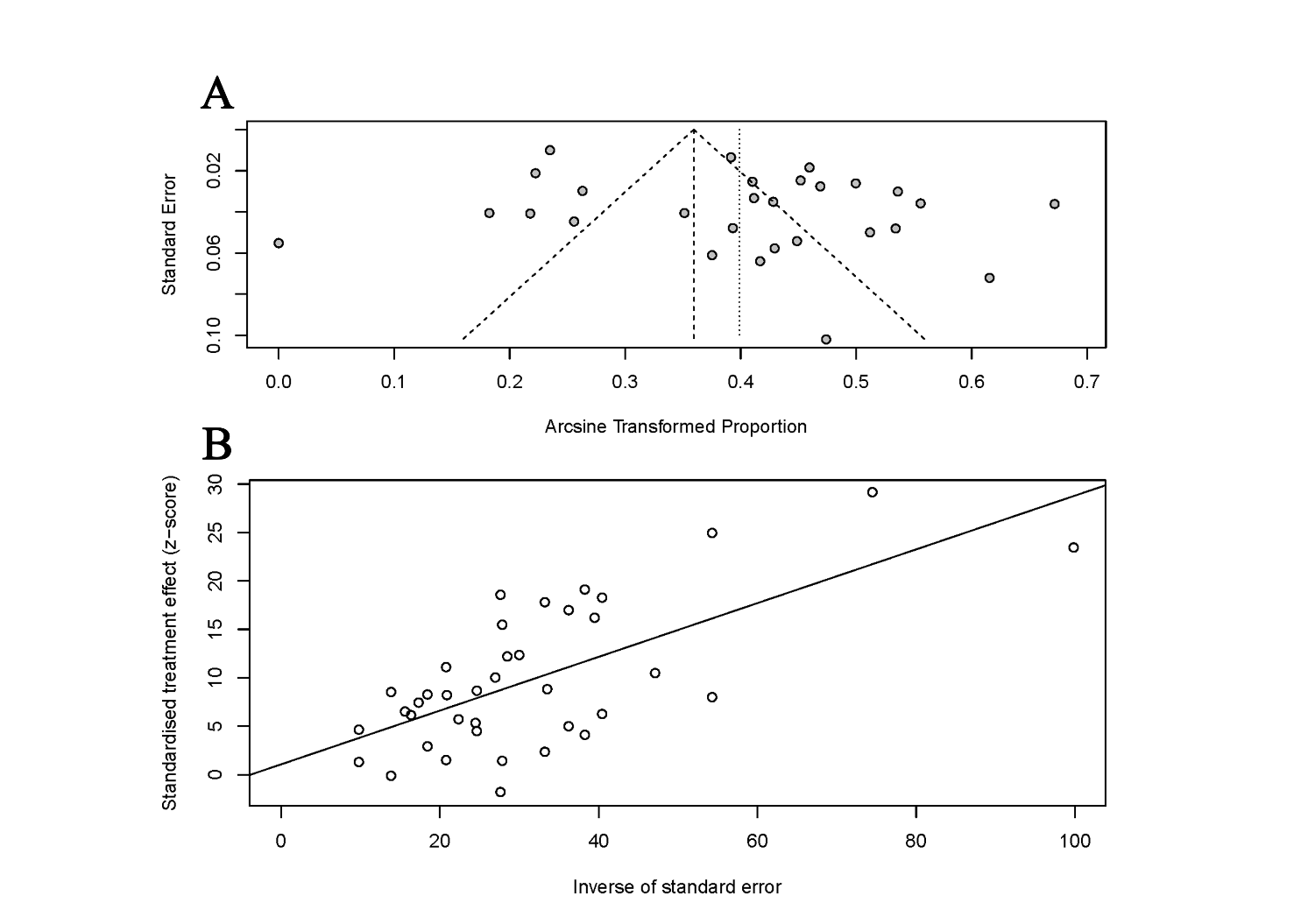
**

**Figure S1.** Please use a single paragraph for each legend and prepare the figures keeping in mind the PDF layout. Publication bias assessment for the meta-analysis of virus tropism prevalence at diagnosis among people living with HIV (PLWH). (A) Visual inspection of the funnel plot. (B) Scatterplot of the Egger’s linear regression test.

**
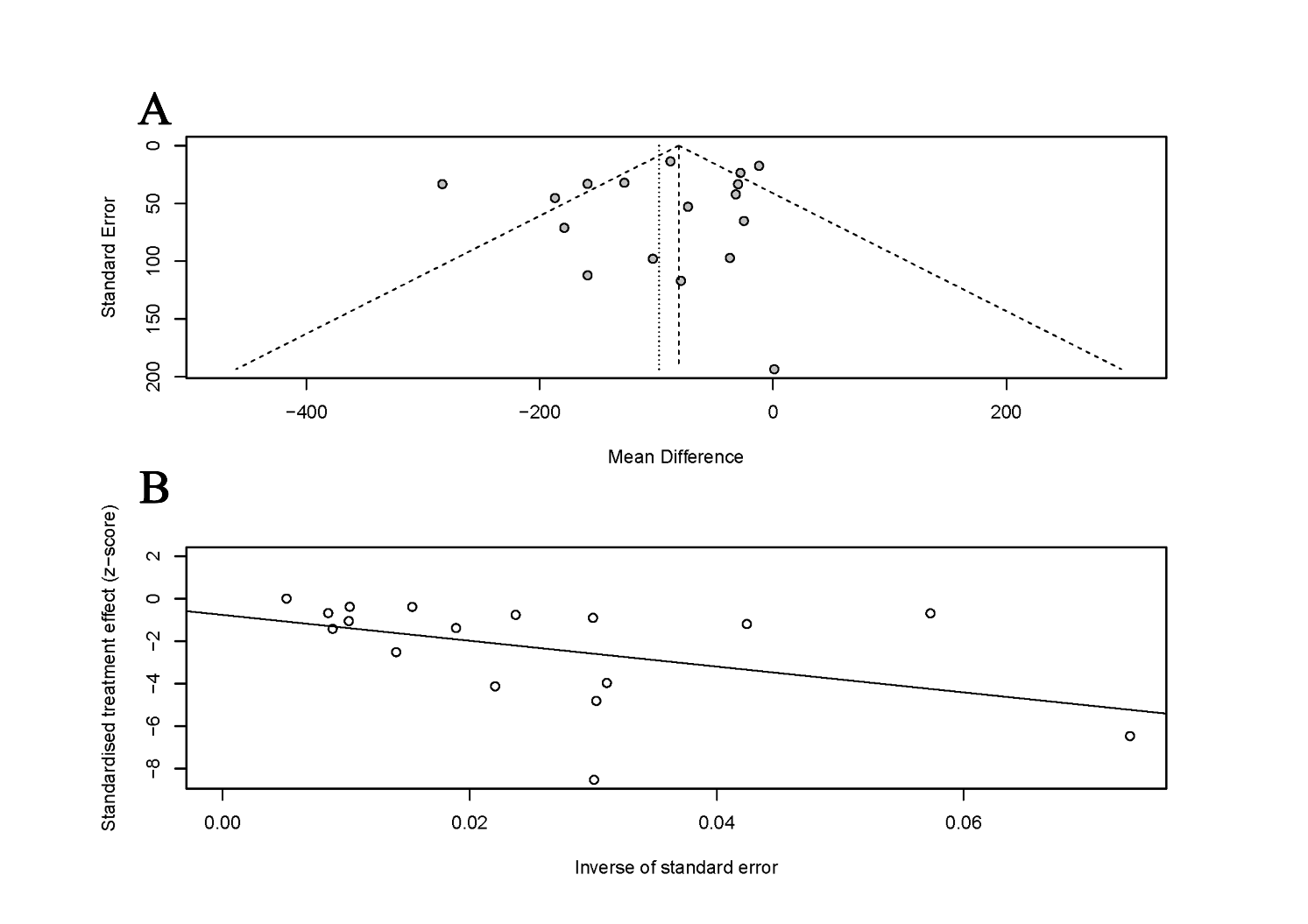
**

**Figure S2.** Publication bias assessment for the meta-analysis of immunological correlates of HIV tropism at diagnosis in people living with HIV (PLWH). (A) Visual inspection of the funnel plot. (B) Scatterplot of the Egger’s linear regression test.

**
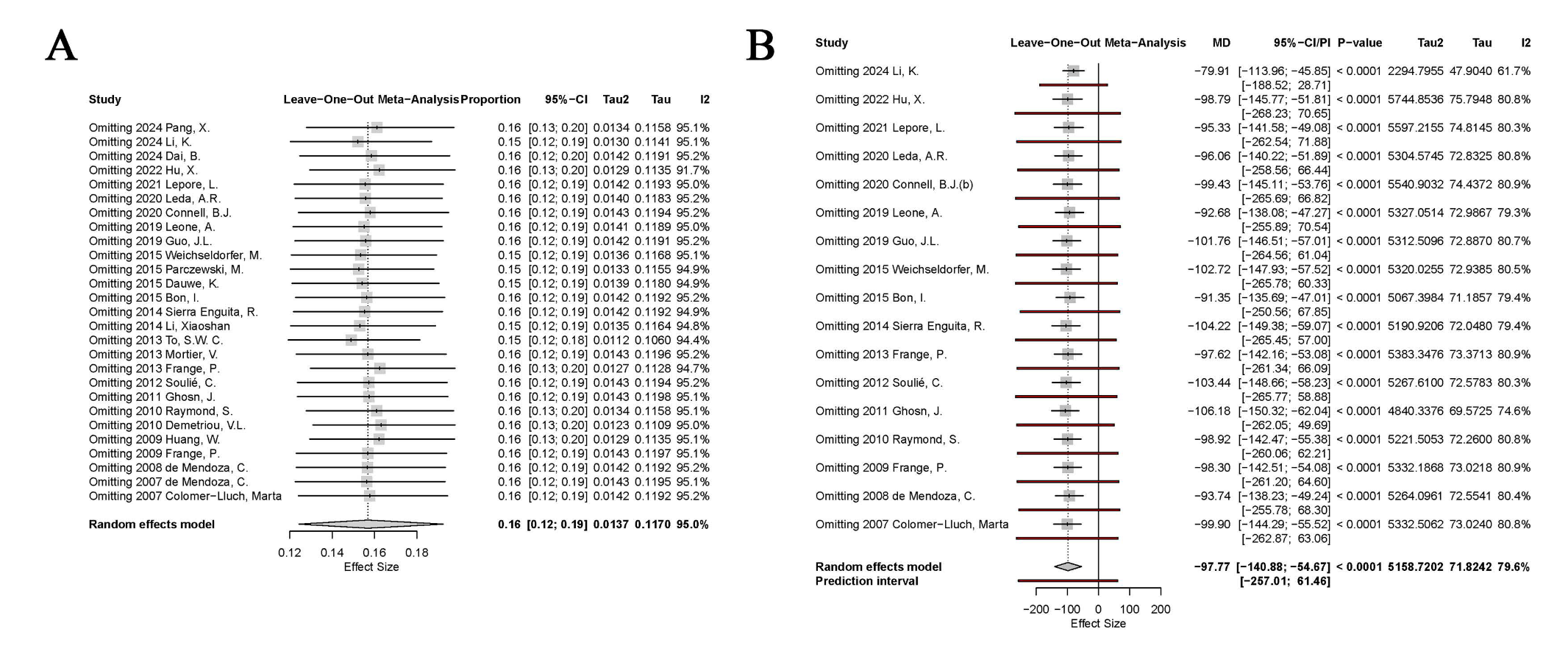
**

**Figure S3.** Forest plots of the leave-one-out sensitivity analysis. (A) For the meta-analysis of viral tropism prevalence at diagnosis. (B) For the meta-analysis of the difference in CD4+ T-cell counts between tropism groups.
